# Lung Cancer and the COVID-19 pandemic: Recommendations from the Brazilian Thoracic Oncology Group

**DOI:** 10.6061/clinics/2020/e2060

**Published:** 2020-06-16

**Authors:** Clarissa Baldotto, Ana Gelatti, Arthur Accioly, Clarissa Mathias, Eldsamira Mascarenhas, Heloisa Carvalho, Lilian Faroni, Luiz Henrique Araújo, Mauro Zukin, Rafael Gadia, Ricardo Mingarini Terra, Rui Haddad, Vladmir Cordeiro de Lima, Gilberto de Castro-Júnior

**Affiliations:** I Oncologia, Instituto D’Or de Pesquisa e Ensino, Rio de Janeiro, RJ, BR; II Grupo Brasileiro de Oncologia Toracica, Porto Alegre, RS, BR; III Sociedade Brasileira de Radioterapia, Sao Paulo, SP, BR; IV Grupo Oncoclinicas, Sao Paulo, SP, BR; V Oncologia D’Or, Salvador, BA, BR; VI Radioterapia, Instituto de Radiologia (INRAD), Hospital das Clinicas HCFMUSP, Faculdade de Medicina, Sao Paulo, SP, BR; VII Hospital Sirio Libanes, Sao Paulo, SP, BR; VIII Instituto Nacional do Cancer, Rio de Janeiro, RJ, BR; IX Instituto COI de Educacao e Pesquisa, Rio de Janeiro, RJ, BR; X Grupo de Oncologia D’Or, Rio de Janeiro, RJ, BR; XI Sociedade Brasileira de Cirurgia Toracica, Sao Paulo, SP, BR; XII Faculdade de Medicina FMUSP, Universidade de Sao Paulo, Sao Paulo, SP, BR.; XIII Academia Nacional de Medicina, Rio de Janeiro, RJ, BR; XIV Oncologia Medica, A.C. Camargo Cancer Center, Sao Paulo, SP, BR

**Keywords:** Coronavirus, SARS-CoV, Lung Cancer

## Abstract

New cases of the novel coronavirus disease 2019 (COVID-19), also known as severe acute respiratory syndrome coronavirus 2 (SARS-CoV-2), continue to rise worldwide following the declaration of a pandemic by the World Health Organization (WHO). The current pandemic has completely altered the workflow of health services worldwide. However, even during this critical period, patients with other diseases, like cancer, need to be properly treated.

A few reports have shown that mortality due to SARS-CoV-2 is higher in elderly patients and those with other active comorbidities, including cancer. Patients with lung cancer are at risk of pulmonary complications from COVID-19, and as such, the risk/benefit ratio of local and systemic anticancer treatment has to be considered. For each patient, several factors, including age, comorbidities, and immunosuppression, as well as the number of hospital visits for treatment, can influence this risk. The number of cases is rising exponentially in Brazil, and it is important to consider the local characteristics when approaching the pandemic. In this regard, the Brazilian Thoracic Oncology Group has developed recommendations to guide decisions in lung cancer treatment during the SARS-CoV-2 pandemic. Due to the scarcity of relevant data, discussions based on disease stage, evaluation of surgical treatment, radiotherapy techniques, systemic therapy, follow-up, and supportive care were carried out, and specific suggestions issued. All recommendations seek to reduce contagion risk by decreasing the number of medical visits and hospitalization, and in the case of immunosuppression, by adapting treatment schemes when possible. This statement should be adjusted according to the reality of each service, and can be revised as new data become available.

## INTRODUCTION

On December 31, 2019, the World Health Organization (WHO) was informed of a high incidence of pneumonia cases in the city of Wuhan, Hubei Province of China. On January 7, 2020, Chinese authorities confirmed the pneumonia outbreak was caused by a new type of virus, which belongs to a family known as Coronaviruses (1). Some of the viruses in the Coronaviruses family already exist in Brazil, and are capable of causing a common cold; however, the coronavirus family also encompasses viruses like SARS and MERS that caused epidemics with a large number of deaths in 2004 and 2012, respectively. The new virus is believed to have been transmitted from another species of animal, bats are currently suspected, to humans (2). On February 26, 2020, the first case of this disease was reported in Brazil, a 61-year-old man who had recently been abroad. Since then, the number of cases grew rapidly, and in early May, the virus death toll in Brazil was over 10,000, with over 600 deaths reported per day (3).

The coronavirus disease 2019 (COVID-19) is the disease caused by the newly identified virus, of the Coronaviruses family - severe acute respiratory syndrome coronavirus 2 (SARS-CoV-2). COVID-19 has evolved rapidly, and based on the increase in the number of cases and fast global spread, the WHO classified the outbreak as a pandemic on March 11, 2020. The COVID-19 pandemic is now one of the world’s most serious public health problems in recent years. This being the case, all health organizations had to drastically rearrange their activities to face the challenges posed by this new disease. These changes are even more challenging when they involve patients with chronic diseases, including cancer. In particular, in the case of patients with suspected or diagnosed lung cancer, there are peculiarities that must be addressed within the context of the current pandemic.

Clinical findings and imaging in the early stages of COVID-19 pneumonia may resemble common findings in patients with lung cancer, like treatment complications (caused by immunotherapy, chemotherapy, radiotherapy, and/or targeted therapies) and other infectious complications (4). Furthermore, patients with suspected or diagnosed lung cancer often undergo invasive thoracic procedures, which increases their risk of complications in case of a SARS-CoV-2 infection. It is important to mention that most are patients with advanced lung cancer for whom decisions regarding non-invasive, psychological and familiar support, and comfort care measures have a critical role.

For these reasons, the Brazilian Thoracic Oncology Group (GBOT) have outlined guidelines that are both consensus-based, representing major cancer treatment medical societies, and localized, taking into account local needs for managing these patients. These guidelines will be updated as and when new relevant information becomes available.

## LUNG CANCER AS A RISK FACTOR FOR COVID-19

Although any individual can be infected by COVID-19, certain populations are more likely to develop complications and severe forms of the disease. Based on previous studies, patients over 65 years of age, living in nursing homes, and suffering from comorbidities, including chronic lung diseases, moderate to severe asthma, heart diseases, immunosuppression conditions, severe obesity (a BMI of 40 or higher), diabetes mellitus, chronic kidney disease on dialysis, and liver disease (5) are more likely to develop severe disease. Consequently, there are greater chances of lung cancer patients falling into a high-risk group. Although all types of neoplasia appear to be associated with high prevalence, morbidity, and mortality from COVID-19, lung cancer represents a specific situation involving cumulative risk factors for COVID-19 complications, including advanced age, significant cardiovascular and respiratory comorbidities, lung damage caused by smoking, impairment of immune function following cancer treatment (or cancer itself), or the frequent use of corticosteroids.

Numerous studies on COVID-19 in cancer patients have confirmed this scenario. Among 1,524 cancer patients admitted to the Department of Radiation and Medical Oncology at Zhongnan Hospital of Wuhan University in Wuhan, China, 0.79% had COVID-19 *versus* 0.37% of the general population of Wuhan over the same study period (OR, 2.31; 95% CI, 1.89-3.02). In the same study, patients with non-small cell lung cancer (NSCLC) seemed to have a higher incidence of COVID-19, especially those over 60 years of age (4.3% *versus* 1.8% in those aged under 60 years with NSCLC) (6). According to another retrospective case study published by a Chinese team, cancer patients (most frequently, advanced lung cancer [28%]) were at higher risk of severe complications and unfavorable outcomes than hospitalized cancer-free patients (7). This same finding was also reported in at least two other analyses. Smoking history also seems to increase the risk of complications by up to 1.4-fold, and by 2.4-fold that of death or need for mechanical ventilation due to COVID-19 (8,9).

During a recent plenary session of the American Association for Cancer Research annual congress on “COVID-19 and Cancer,” an international team led by Dr. Marina Garassino presented initial data from TERAVOLT (Thoracic cancERs internAtional coVid 19 cOLlaboraTion) a global registry gathering information on patients with thoracic cancers infected with COVID-19 - in which a 34.6% mortality rate (66/191) among patients with thoracic cancer was reported. In addition, the most common complications were pneumonia and pneumonitis (79.6%; 125/157), acute respiratory distress syndrome (26.8%; 42/157), multiple organ failure (7.6%; 12/157), and sepsis in (5.1%; 8/157). The cause of death in most of these patients was attributed to COVID-19 and not to cancer itself (10). For these reasons, lung cancer patients must be considered to be at greater risk of developing COVID-19 complications, regardless of the cancer staging.

## RECOMMENDATION OF LUNG CANCER MANAGEMENT ACCORDING TO DISEASE STAGE

Decisions about procedures, work-up, and treatment must be tailored by factors like disease stage, molecular and histology diagnosis, patient clinical status, local infrastructure, and pandemic characteristics. In this regard, the following recommendations were adopted by the American College of Surgeons classification of phased units, based on disease burden and available facilities and resources (Table 1) (11).

### MANAGEMENT DURING DIAGNOSIS AND STAGING

Given the high aggressiveness and mortality of lung cancer, decisions to postpone diagnostic procedures must be evaluated very carefully on a case-by-case basis, and patient and family expectations should be considered in the decision. The risks of medical visits and procedures can vary, and depend on social isolation, exposition to contaminated patients or health staff in outpatient and inpatient units, invasive procedures to the airways and thorax, hospitalization, and immunosuppression. In all situations, it is recommended to closely observe the current status and changes in the scenario of individual institutions in the face of the pandemic. Whenever possible, patients should be transported to Phase 1 units and the following measures evaluated:

1. Limit the number of invasive procedures and hospitalizations when there is no harm to the conduction of the case. For instance, mediastinoscopy and surgery could be performed at the same time, and diagnostic biopsies could be omitted in highly suspicious lesions before surgery.
2. Avoid procedures with airway manipulation, such as diagnostic bronchoscopy, endoscopic bronchoscopy (EBUS) for diagnosis and staging, echoendoscopy, and pulmonary function tests (spirometry).
3. Prefer percutaneous procedures for diagnosis and staging, performed in an outpatient or day hospital facility.
4. Perform PET-Computed Tomography (CT) scan only when it can potentially modify staging. For instance, multiple imaging exams could be avoided in patients already diagnosed with stage IV disease.
5. Concentrate staging exams on the same day.
6. Option for imaging follow-up is possible in cases of low or intermediate risk: a) lesions with predominant ground glass opacities (<50% solid area); b) solid nodule smaller than 2 cm; c) pleural-based solid nodule <5 mm; d) benign morphology; f) known volume doubling time >600 days (11);
7. Lung cancer screening should be deferred until the COVID-19 pandemic resolves (12).

### INITIAL DISEASE MANAGEMENT

There is still paucity of data addressing initial lung cancer during COVID-19. In this subgroup, age was an important risk factor for complications, and the treatment complication rates appear to be higher in patients with advanced disease. Therefore, when approaching a patient with initial disease, we must consider age, the presence of other comorbidities, contagion risk, potential treatment-related or primary immunosuppression, use of ventilation equipment, and intensive care unit (ICU) beds. In this sense, we recommend, whenever possible, to transfer the patient to Phase 1 units and evaluate the following measures:

1. Discuss stereotactic ablative radiotherapy (SABR) in patients with stage I and II disease, especially if ≥70 years of age and at higher surgical risk.
2. Prefer hypofractionated radiation therapy when possible. For peripheral located lesions, treatment may be performed over 1-3 fractions, for central located lesions in five fractions, and for ultra-central located lesions in eight fractions.
3. Avoid open surgeries (thoracotomy) or any procedure that could increase the postoperative period and complications.
4. Minimally invasive techniques are highly recommended, like video assisted (VATS) or robotic video assisted techniques (R-VATS). During the surgical approach, CO_2_ insufflation is possible, avoiding gas leakage through the portals, and taking extra care at the end of the procedure, before removing the surgical specimen, to filter gas from the cavity through antiviral filters. Bipolar energy instruments are recommended, to reduce smoke formation and aerosolization. Connect an antiviral filter or other suitable device to the drainage system, especially if there is an air leak.
5. Use of personal protective equipment (PPE) by authorized professionals during the surgical procedure.
6. Perform chest CT scan 24-48 hours before surgery, to assess the presence of suspected lesions for COVID-19 (recommended).
7. Collect nasal swab to perform PCR to test for SARS-CoV-2 before the procedure (recommended).
8. Surgical approaches that should not be delayed at this time, unless only Phase 3 units are available:
  - Solid or predominantly solid lesions (>50%) in patients with NSCLC with negative lymph nodes.
  - Suspected lesions for lung cancer larger than 2.0 cm with negative lymph nodes.
  - Mediastinoscopy or surgical procedures for staging; for example, videothoracoscopy to assess pleural effusion.
9. Surgical approaches that may be postponed:
  - Indolent histologies (carcinoid tumor, lepidic adenocarcinoma, with pure ground glass opacity).
  - Resectable small cell lung cancer (SCLC) patients - start with neoadjuvant chemotherapy.
10. Do not indicate adjuvant chemotherapy in CPNPC stage <IB (T2aN0).
11. In candidates for perioperative chemotherapy (CT), prefer neoadjuvant chemotherapy (recommended schemes with less risk of hospitalization and number of visits to the treatment center: Non-squamous histology, Cisplatin/Pemetrexed; squamous histology: cisplatin/docetaxel).
12. Use granulocyte colony-stimulating factor (G-CSF) as primary prophylaxis to reduce risk of hospitalization during neoadjuvant or adjuvant CT if risk of febrile neutropenia is ≥10%.
13. Consider adjuvant treatment with gefitinib 250 mg VO for 2 years, for elderly operated patients with a sensitivity EGFR mutation (exon 19 deletion or L858R exon 21 mutation) who have indication of adjuvant CT and higher risk of COVID-19 complications.
14. Prophylactic cranial irradiation for SCLC should be discussed, and may be postponed for up to 2-3 months.

### LOCALLY ADVANCED DISEASE MANAGEMENT

Patients with locally advanced disease may present in different stages and situations, and decisions should be made on a case-by-case basis. All previous recommendations regarding surgical and staging procedures should be followed. Since locally advanced lung cancer is a high-risk disease, as a general rule, treatments should not be postponed, but adapted whenever possible, to deal with challenges brought by the pandemic. We must take into account the presence of other comorbidities, age, performance status (PS), and treatment-related or primary immunosuppression, and try to reduce the contagion risk, number of visits to the treatment center, hospitalizations, use of ventilation equipment, and ICU beds. We recommend, whenever possible, to transfer patients to Phase 1 hospitals and evaluate the following measures:

1. Keep multidisciplinary decisions through web-based tumor boards.
2. For candidates of perioperative CT, prefer a neoadjuvant approach (recommended schemes with less risk of hospitalization and number of visits to the treatment center: Non-squamous histology, cisplatin/pemetrexed; squamous histology: cisplatin/docetaxel).
3. Candidates with concomitant CT and radiotherapy should not have treatment suspended or postponed. Instead, CT schemes with less infusion days and less risk of hospitalization or immunosuppression should be considered. Radiotherapy should be started on the first day of CT infusion (to avoid more CT cycles). Furthermore, the standard RT dosage for locally advanced disease is 60 Gy in 30 fractions, but hypofractionated schemes can be adopted, respecting equivalent efficacy dosage and dose-volume predictors and constraints for toxicity.
4. Immunotherapy maintenance with durvalumab can be prescribed, and an adapted schedule might be considered (durvalumab 1500 mg IV every 4 weeks). This recommendation is not available as drug package insert, but we consider that there is sufficient security and efficacy data published (13).
5. Adjuvant radiotherapy for pathologic stage N2 disease (pN2) should be individualized.
6. Adjuvant radiotherapy for N0 disease post neoadjuvant treatment (ypN0) should not be recommended.
7. Prophylactic cranial irradiation for SCLC should be discussed, and may be postponed for up to 2-3 months.
8. Consolidation thoracic irradiation for SCLC should not be routinely indicated.
9. Use G-CS as primary prophylaxis, to the reduce risk of hospitalization during CT if risk of febrile neutropenia ≥10%.

### ADVANCED DISEASE MANAGEMENT

Although patients with advanced disease are at greater risk of complications from COVID-19, due to the severity of the neoplasia, treatments should generally not be postponed. We must consider the presence of other comorbidities, age, and PS, and try to reduce contagion risk, decrease the hospitalizations and the number of visits to the treatment center, reduce immunosuppression, and reduce use of equipment and ICU beds in hospitals. We recommend, whenever possible, to transfer patients to Phase 1 hospitals and evaluate the following measures (12):

1. Prefer to start targeted therapy over CT/immunotherapy (IO), whenever possible. In patients already undergoing CT with partial response, a change to targeted therapy should be considered.
2. For patients with high PD-L1 expression (≥50%): consider using IO as monotherapy, with adapted dosage (pembrolizumab 400 mg IV every 6 weeks).
3. For patients undergoing IO, adapt the dosage (Nivolumab 480 mg IV every 4 weeks; Pembrolizumab 400 mg IV every 6 weeks).
4. For patients undergoing maintenance CT (pemetrexed) and IO, consider pemetrexed suspension and maintenance of IO alone with an adapted dosage (pembrolizumab 400 mg IV every 6 weeks).
5. Avoid prescribing a third or fourth line of chemotherapy in patients with comorbidities.
6. Use G-CSF as primary prophylaxis to reduce the risk of hospitalization during CT, if the risk of febrile neutropenia is ≥10%.
7. Adopt home administration for subcutaneous medications if feasible.
8. Postpone infusion of bisphosphonates (acid zolendronic every 3 months).
9. Prefer darbepoetin alfa 500 mg SC every 3 weeks for the treatment of anemia secondary to CT. Avoid blood transfusions.
10. When possible, postpone local treatment of oligometastasis. Prefer SABR and avoid surgical procedures in these cases.
11. Brain metastases: prefer radiotherapy to surgery, and hypofractionated radiotherapy when possible. Avoid radiation therapy or surgery if effective targeted therapy is available and the disease is oligo- or asymptomatic.
12. Prophylactic CNS radiotherapy for SCLC should not be indicated. Consider follow-up with brain magnetic resonance imaging (MRI).

### ADVANCED DISEASE - PALLIATIVE CARE MANAGEMENT

Patients with advanced lung cancer in palliative care are a major challenge in the face of the COVID-19 pandemic. In this scenario, we must reduce contagion risk and family exposure, promote social isolation, try to reduce the use of ventilation equipment and ICU beds in hospitals, and provide psychological support to patients and their family. Whenever possible, we recommend transferring the patient to Phase 1 hospitals and implementing the following measures:

1. Discuss, for patients in exclusive palliative care, living wills and advance directives for medical decisions.
2. Define in medical records the level of support that can be administered in an emergency.
3. Prefer home care when possible.
4. Allow and provide virtual contact with family and professionals for psychological support if isolation is recommended.

### MANAGEMENT OF PATIENTS IN FOLLOW-UP

The follow-up of lung cancer patients depends on the histological subtype (SCLC *versus* NSCLC) and the tumor stage (I and II *versus* III *versus* IV). In the current SARS-CoV-2 pandemic scenario we should seek to reduce the risk of patient contamination, promote social isolation, efficiently detect COVID-9 cases among cancer patients, monitor the patient’s clinical status, minimize the risk of cancer progression, guarantee timely communication with the attending physician and the patient’s cancer care team, and provide psychological support and reassurance (14).

The recommendations for lung cancer patients follow-up are as follows:

1. Stage I NSCLC patients with no new symptoms: postpone follow-up image work-up and visits.
2. Stage II and III NSCLC patients treated with curative intent (surgery or concurrent CT and radiotherapy for stage III disease) whose treatment was completed over more than 1 year, without evidence of disease activity and with no new symptoms: postpone image work-up up to 1 year. Maintain, if available, telemedicine follow-up visits to check clinical status.
3. Stage II and III NSCLC patients treated with curative intent (surgery), or stage III patients treated with CT and radiotherapy, whose treatment was completed in less than 1 year, with no new symptoms: postpone follow-up image work-up and visits up to 6 months.
4. Stage II and III NSCLC patients treated with palliative intent, with no new symptoms: postpone follow-up image work-up and visits up to 6 months. Maintain, if available, telemedicine follow-up visits every 3 months to check clinical status.
5. Stage I to III SCLC patients treated with curative intent, whose treatment was completed in less than 2 years, without evidence of disease activity at last follow-up (if less than 6 months) and with no new symptoms: postpone follow-up image work-up and visits up to 6 months. Maintain, if available, telemedicine follow-up visits every 3 months to check clinical status.
6. Stage I to III SCLC patients treated with curative intent, whose treatment was completed over more than 2 years, without evidence of disease activity at last follow-up (if less than 6 months) and with no new symptoms: postpone follow-up image work-up and visits up to 1 year. Maintain, if available, telemedicine follow-up visits every 6 months to check clinical status.
7. Previously treated stage IV NSCLC or SCLC, but not receiving active treatment currently, with no new symptoms: postpone follow-up image work-up and visits up to 3 months. Limit image work-up (*i.e.* prefer PET-TC scan over CAT scans and bone scintigraphy). Repeat brain MRI only if the patient is symptomatic. Maintain, if available, telemedicine follow-up visits every 6 weeks to check clinical status.
8. Stage IV NSCLC or SCLC currently receiving active treatment, with no new symptoms: postpone image work-up up to 3-4 months. Limit image work-up (i.e. prefer PET-TC scan over CAT scans and bone scintigraphy). Repeat brain MRI only if the patient is symptomatic. Maintain follow-up visits every other cycle if patient is receiving chemotherapy. If the patient is being treated with tyrosine kinase inhibitors or ICIs, postpone follow-up visits up to 3-4 months. Maintain, if available, telemedicine follow-up visits every 6 weeks to check clinical status.
9. Patients with brain metastasis: Repeat brain MRI only if symptomatic.

### FURTHER RECOMMENDATIONS

1. Preferably, patients should be contacted on the day prior to their visit and questioned whether they, or any of their close contacts, have had any close contact with COVID-19 confirmed patients, or developed COVID-19 associated symptoms in the last 14 days. This questioning should be repeated upon their arrival at the clinic.
2. If the patient has/had COVID-19 associated symptoms or contact with COVID-19 infected individuals, their visit should be postponed. If the symptoms are mild, they should be tested for SARS-CoV-2 infection (PCR - nasal swab), put into domiciliary isolation and followed up by telemedicine for 14 days, before re-starting treatment. If symptoms are moderate to severe, the patient should be hospitalized.
3. Limit number of companions to one, and restrict, if possible, access of companions to the clinic. Companions should also be interrogated about COVID-19 associated symptoms and whether they have had contact with a COVID-19 confirmed case in the last 14 days.
4. Patients who tested positive for SARS-CoV-2 must remain isolated for 14 days after the start of symptoms or after the date of the test (if asymptomatic). Depending on the treatment and the severity of symptoms, the treatment re-start should be delayed by up to 4 weeks or even changed. Preferably, a new PCR from nasal swab should be performed before treatment re-initiation. Patients already on course of radiotherapy should not have treatment interrupted unless necessary. Safety precautions must be taken.
5. All patients and companions should wear masks while in the clinic.
6. Health care providers must use adequate PPE.
7. Intravenous catheter maintenance should be scheduled every 12 weeks if not under use.
8. Telemedicine consultations should be preferred when feasible. Telemedicine consultations should be avoided in patients with worsening or new symptoms, for first time visits, and for first follow-up visit after treatment start.
9. Patients who develop treatment complications should refrain from being hospitalized in Phase 2 and 3 hospitals.
10. Influenza vaccination should be encouraged, as well pneumococcic vaccination.
11. Patients suspected to have drug-induced or immune-related pneumonitis and concomitant COVID-19 must not have steroid initiation postponed if deemed necessary by the treating physician.

## FINAL CONSIDERATIONS

COVID-19 is a very recently described disease. These are general recommendations, based on actual information available. All cases should be discussed on an individual basis, and multidisciplinary meetings through web conferences are highly recommended. As mentioned previously, all recommendations should be adjusted according to the reality of each service and epidemiological issues. New and revised recommendations may arise at any time, as new data become available.

## AUTHOR CONTRIBUTIONS

All authors were fully involved in the production of this manuscript and participated in the design, acquisition and discussion of data. Baldotto C drafted the manuscript. All of the other authors contributed to specific parts and critically reviewed the manuscript. All of the authors read the final version of the manuscript and agreed with the submission.

## Figures and Tables

**Table 1:** Facilities classification for elective case triage for cancer care.

Phase	Facility and epidemiogical characteristics
Phase 1	Few COVID-19 patients, hospital resources not exhausted, institution still has ICU vent capacity, and COVID trajectory not in rapid escalation phase
Phase 2	Many COVID-19 patients, ICU and ventilator capacity limited, OR supplies limited, or COVID trajectory within hospital in rapidly escalating phase
Phase 3	Hospital resources are all routed to COVID-19 patients, no ventilator or ICU capacity, OR supplies exhausted
